# Development of a reliable surgical quality assurance tool for gastrectomy in oncological trials

**DOI:** 10.1007/s10120-024-01503-8

**Published:** 2024-05-18

**Authors:** A. Harris, J. B. Butterworth, P. R. Boshier, S. Mavroveli, B. Vadhwana, C. J. Peters, B. W. Eom, C.-C. Yeh, S. Mikhail, M. Sasako, Y.-W. Kim, G. B. Hanna

**Affiliations:** 1 Department of Surgery and Cancer, Imperial College London, 7th Floor Commonwealth Building, Hammersmith Hospital, Du Cane Road, London, W12 0HS UK; 2 Department of Upper Gastrointestinal Surgery, Barking Havering and Redbridge University Hospitals NHS Trust, London, UK; 3 Center for Gastric Cancer, National Cancer Center, Seoul, Republic of Korea; 4 Department of Surgery, National Taiwan University Hospital, Taipei City, Taiwan; 5 Department of General Surgery, Cairo University, Cairo, Egypt; 6 Department of Surgery, Yodogawa Christian Hospital, Osaka, Japan

**Keywords:** Gastric cancer, Gastrectomy, Surgical quality assurance

## Abstract

**Background:**

Despite its recognized importance, there is currently no reliable tool for surgical quality assurance (SQA) of gastrectomy in surgical oncology. The aim of this study was to develop an SQA tool for gastrectomy and to apply this tool within the ADDICT Trial in order to assess the extent and completeness of lymphadenectomy.

**Methods:**

The operative steps for D1+ and D2 gastrectomy have been previously described in the literature and ADDICT trial manual. Two researchers also performed fieldwork in the UK and Japan to document key operative steps through photographs and semi-structured interviews with expert surgeons. This provided the steps that were used as the framework for the SQA tool. Sixty-two photographic cases from the ADDICT Trial were rated by three independent surgeons. Generalizability (G) theory determined inter-rater reliability. D-studies examined the effect of varying the number of assessors and photographic series they rated. Chi-square assessed intra-rater reliability, comparing how the individual assessor’s responses corresponded to their global rating for extent of lymphadenectomy.

**Results:**

The tool comprised 20 items, including 19 anatomical landmarks and a global rating score. Overall reliability had G-coefficient of 0.557. Internal consistency was measured with a Cronbach’s alpha score of 0.869 and Chi-square confirmed intra-rater reliability for each assessor as < 0.05.

**Conclusions:**

A photographic surgical quality assurance tool is presented for gastrectomy. Using this tool, the assessor can reliably determine not only the quality but also the extent of the lymphadenectomy performed based on remaining anatomy rather than the excised specimen.

## Introduction

Surgical quality assurance (SQA) is a formal process of ensuring the quality of surgical interventions, most commonly in the context of a randomized controlled trial (RCT). To be considered definitive, the SQA must be demonstrably robust. It encompasses trial entry criteria for individual surgeons and centers, standardization of surgical technique, and performance monitoring. There is evidence that SQA in surgical RCTs for gastro-esophageal cancer reduces variation in lymph node harvest, in-hospital mortality, and loco-regional disease recurrence [1].

While there is broad acceptance of the principles and importance of SQA, it has not always been robustly applied within surgical trials. The absence of adequate SQA in previous trials may have ultimately influenced their final outcomes [2–5]. Multiple challenges to the implementation of SQA in trials have been reported, including a failure to adequately standardize surgical interventions and the lack of adequate tools to grade adherence to the expected standard [6].

This research paper describes the development of a reliable quality assurance system for gastrectomy performed in the context of the ADDICT Trial, a multicenter RCT investigating D1+ versus D2 distal gastrectomy as tailored treatments for the surgical management of stage 1 and 2 gastric cancer [7, 8].

## Methods

The SQA process described herein has evolved from previous research published by our research group [9–12]. Steps include: (i) hierarchical task analysis (HTA); (ii) consensus agreement of the HTA to confirm its face and content validity; (iii) development of a tool with descriptors for the operative process (e.g., safety and efficiency) and/or outcome (e.g., quality of the surgical end product); (iv) assessment of (inter and intra-rater) reliability of the tool; and (v) implementation within a clinical trial.

## Structured observations and semi-structured interviews

Two researchers (AH and PB) attended three high-volume centers in the UK and one in Japan where D2 gastrectomy is regularly performed. They observed four senior surgeons, including two world-renowned experts, perform more than fifty gastrectomies. Structured observations were recorded in a research diary, along with notes made during formal and informal interviews with operating surgeons and their teams. These were used to consolidate and supplement research findings from other data sources including structured field notes, surgeons’ operation notes, peri-operative clinical protocols, the published literature, and operative textbooks. Key operative steps were evidenced by photographs or videos, with the appropriate ethical approval and consent in place. Images were stored on Imperial College, password-protected, devices. Semi-structured interviews were digitally audio-recorded with the intention to perform subsequent thematic analysis [18]. A semi-structured interview with MS was deemed to ‘stand-alone’ given his recognized status as a world expert in D2 gastrectomy.

## Hierarchical task analysis and consensus agreement for D2 gastrectomy

**Fig. 1:**
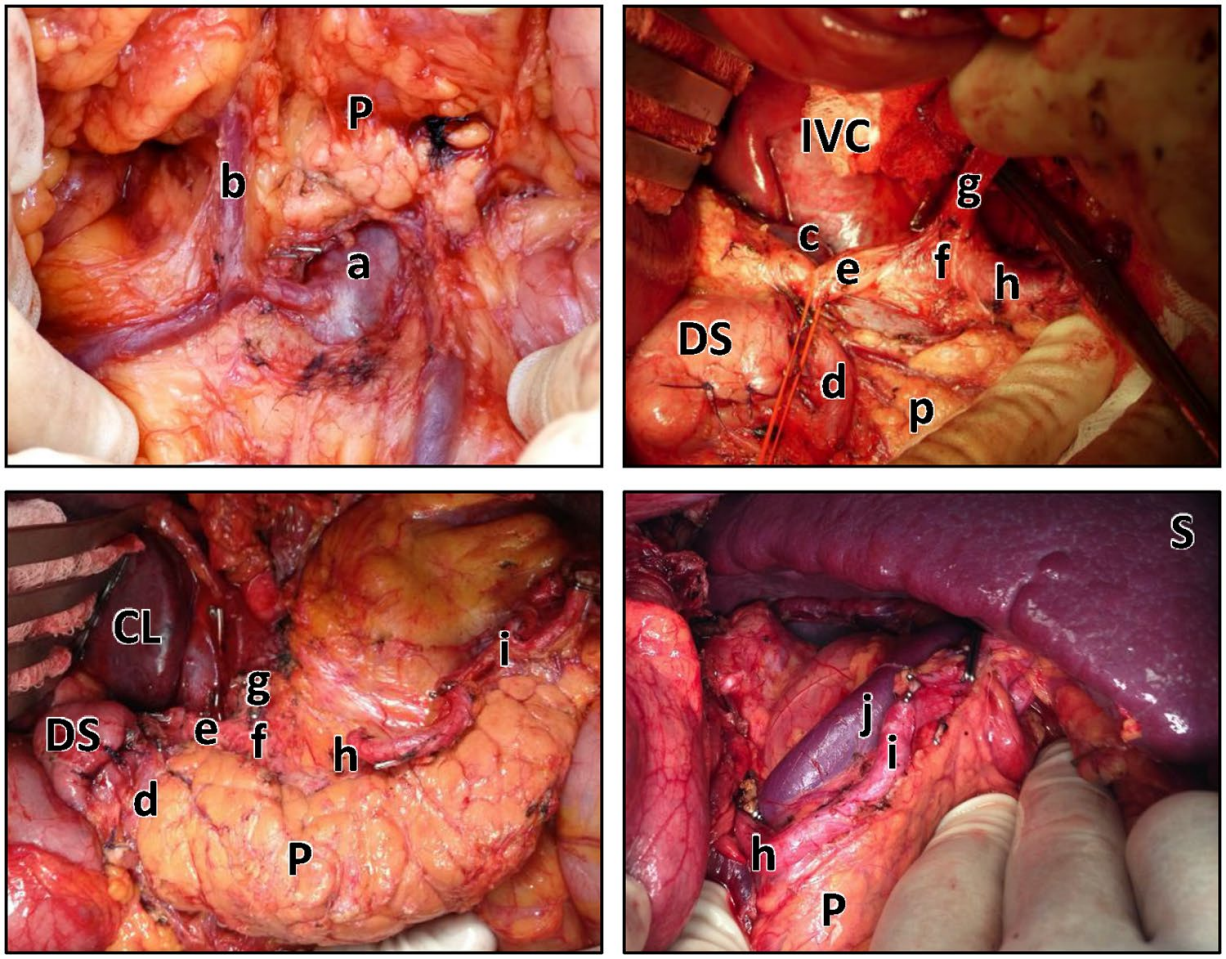
Example photographs for D2 lymphadenectomy from the HTA manual. (a) superior mesenteric vein; (b) right gastroepiploic vein; (c) portal vein; (d) gastroduodenal artery; (e) common hepatic artery; (f) coeliac axis; (g) left gastric artery; (h) proximal splenic artery; (i) distal splenic artery; (j) splenic vein; (p) pancreas; (ds) duodenal stump; (IVC) inferior vena cava; (CL) caudate lobe of the liver, and; (S) spleen

The operative steps for D2 gastrectomy have been widely reported and are accepted by surgeons. It was, therefore, deemed inappropriate to undertake a formal HTA with Delphi consensus as part of this study. Nevertheless, an HTA was written and tested for accuracy based on the evidence collated and described above, until no further changes were identified. The final HTA comprised the anatomical landmarks for D2 gastrectomy, triangulated from the existing literature [13–17], direct structured observations, and semi-structured interviews with peer-identified expert gastric cancer surgeons working in specialist centers in the UK and Japan. This document was illustrated with photographs that were taken to demonstrate the key steps and anatomy for a pancreas and spleen preserving D2 gastrectomy. Example photographs of the expected standard for D2 lymphadenectomy can be found in Fig. 1.

A panel of senior gastric cancer surgeons from the UK, Japan and South Korea (CCY, SM, GBH, MS, and YWK) provided verbal and written feedback on the content of the HTA. For the reasons outlined above, no formal measure of consensus agreement was deemed necessary.

## Development of photographic assessment tool

Surgical outcomes are commonly assessed clinically, radiologically and through histopathology of the excised specimen. The SQA requirements of the ADDICT trial, however, necessitated a shift in focus to confirm both the extent and completeness of lymphadenectomy, including an objective assessment of the amount of lymphatic tissue remaining around an anatomical landmark that should have been cleared in a D1+ or D2 gastrectomy.

The operative procedure for gastrectomy was, therefore, divided into two steps, namely resection and reconstruction. The extent of the lymphadenectomy was categorized according to D1 (perigastric lymph nodes), D1+ (D1 plus common hepatic, coeliac and proximal splenic lymphatic tissue) and D2 (D1+ in addition to hepatic artery proper and distal splenic artery lymphatic tissue).

**Fig. 2:**
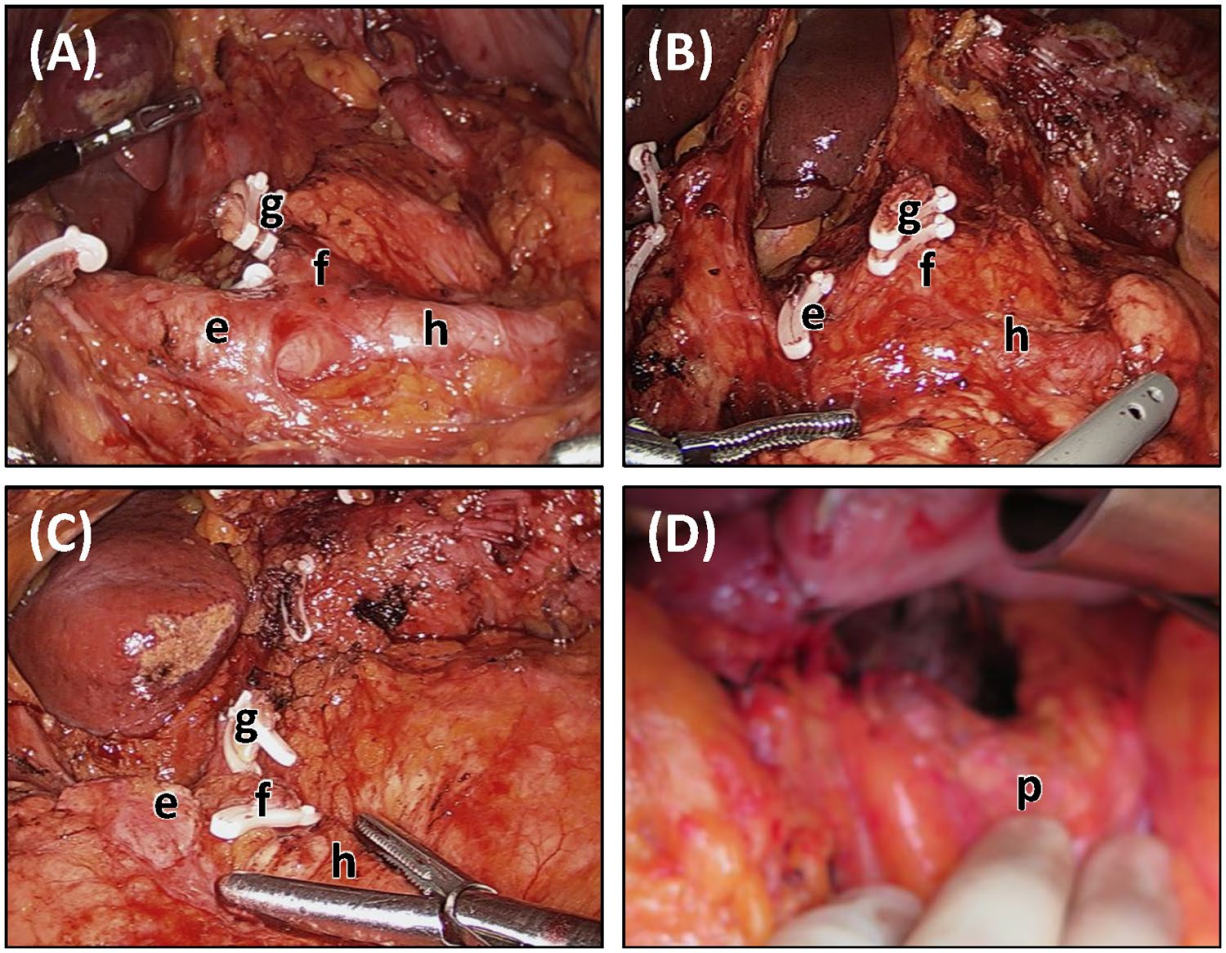
Example photographs demonstrating the different classifications relating to the extent of lymphadenectomy for the coeliac axis and related arteries. (**A**) Compete lymphadenectomy; (**B**) incomplete lymphadenectomy; (**C**) lymphadenectomy not performed, and; (**D**) unable to rate due to insufficient evidence (blurred image). (e) Common hepatic artery; (f) coeliac axis; (g) left gastric artery; (h) proximal splenic artery, and; (p) pancreas

The focus of this research was on the resection, with nineteen anatomical landmarks extracted from within the HTA. These were combined with six descriptors for the lymphadenectomy, updated from the three previously published in the esophagectomy tool [11]. The three additional categories, identified following feedback from assessors using the esophagectomy tool, included: *not applicable* (e.g., lymph node stations 2, 4sa and 11d, which can be omitted in sub-total gastrectomy [14–17]); *insufficient evidence* (e.g., when evidence was submitted but could not be assessed due to blurring or an obstructed field of view), and; *absent data* (e.g., when no relevant evidence was submitted for assessment). Example photographs demonstrating the different classifications relating to the extent of lymphadenectomy for the coeliac axis and related arteries are shown in Fig. 2. A white space section was included for assessors to comment on potentially important factors such as anatomical variations. The anatomical landmarks and proposed changes to the SQA tool were confirmed by a round table discussion with a panel of senior upper gastrointestinal cancer surgeons (CCY, SM and GBH).

The reconstruction section of the tool was adapted from the oesophagectomy tool but did not form the central focus of this new research. It assessed the formation of the esophago/gastro-jejunal and jejuno-jejunal anastomoses with specific descriptors given for each.

**Fig. 3:**
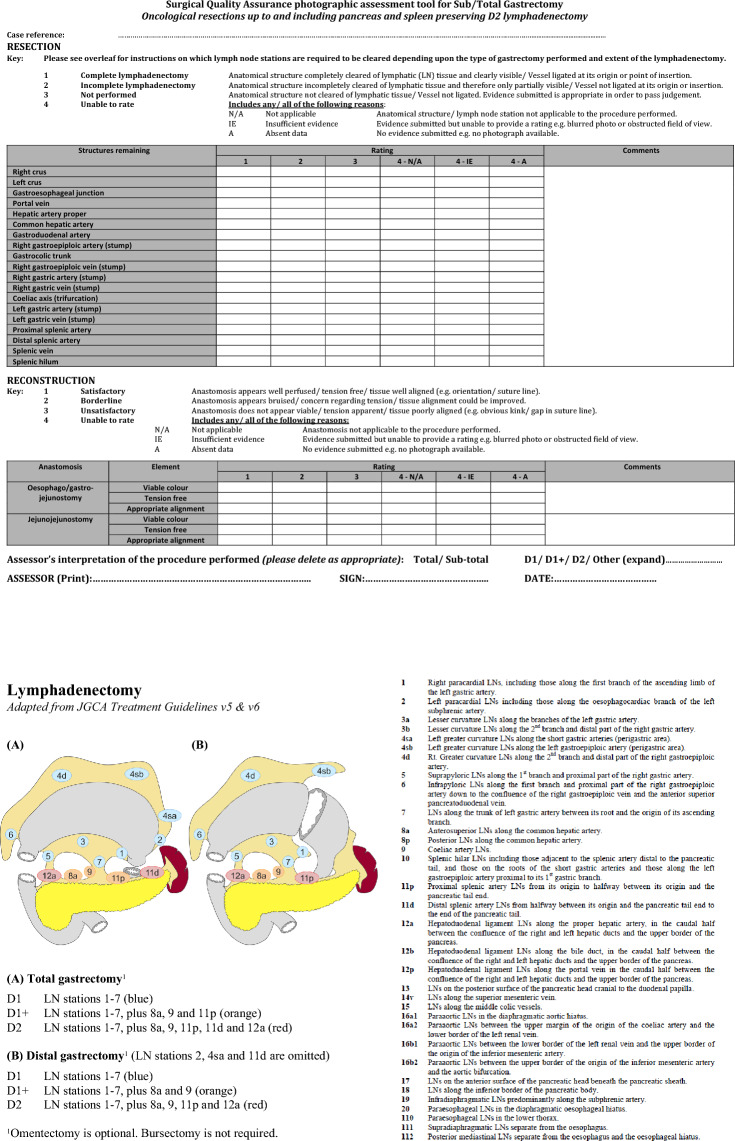
Photographic assessment tool for D2 gastrectomy

A final global rating score summarized the assessors’ overall interpretation of the procedure performed as D1, D1+, or D2 gastrectomy. The final photographic assessment tool is shown in Fig. 3.

## Reliability assessment of the photographic assessment tool

Three independent assessors based in the UK, South Korea and Japan with considerable experience of gastric cancer surgery were invited to participate in the reliability assessment of the photographic tool. All three assessors attended a formal training session, led by the primary researcher. This provided them with an opportunity to clarify the descriptors used in the tool and calibrate their assessments in line with those of their counterparts during example case discussions. The content and duration of the training session was guided by the assessors themselves and only concluded once participants were satisfied that they understood how the tool should be used. Follow up sessions, proposed to address flaws or individual issues, were declined given assessor satisfaction with the tool.

Each assessor was provided with an encrypted memory stick loaded with sixty-two anonymized series of photographs from the ADDICT trial. Their individual responses were recorded on hard copies of the tool, which were subsequently uploaded into a master spreadsheet using Microsoft Excel (Ver. 16.66, Microsoft, Redmond, WA).

## Statistical analysis

Reliability of the photographic assessment tool was evaluated using Generalizability (G) theory [19]. A benefit of G-theory over classical test theory is its ability to assess multiple aspects of reliability (e.g., inter-rater, inter-test, and intra-test) within the same model. Further analysis in the form of a decision (D) study was performed to determine the combination of components that yielded the maximum generalizability. G-string software [20] was used to conduct the generalizability theory, inter-rater reliability, and internal consistency analysis. Cronbach alpha for internal consistency was performed using IBM SPSS statistics (Ver.24, SPSS Inc., Chicago, IL) as part of cross-validation [21]. Chi-square Test of Association was also used to assess if the individual assessor’s responses corresponded to their global rating for the extent of the lymphadenectomy. Phi and Cramer’s V [22] were used to demonstrate the strength of association between assessors’ ratings.

## Results

### Data collection and analysis

Sixty-two photographic series of individual gastrectomies performed within the ADDICT trial were made available for assessment by three independent and experienced gastric cancer surgeons. The number of photographs submitted per photographic series ranged from four to nine, with a median of six photographs per gastrectomy. No photographs of the reconstruction were provided to the assessors and so the reconstruction section of the tool was not formally assessed.

The resection section of the tool comprised 20 items, consisting of 19 anatomical landmarks and the single global rating score. In total, 186 assessment forms (comprising 62 photographic series rated by three assessors) of the 20-item photographic assessment tool were analyzed.

Of a combined 3,720 data entry points for all 62 resections, 80 were *absent* (2.2%), 418 (11.2%) had *insufficient evidence* to rate, and 353 (9.5%) were rated as *not applicable*, indicating that assessors could see that a distal gastrectomy had been performed.

## Generalizability theory results for the gastrectomy photographic assessment tool

**Fig. 4:**
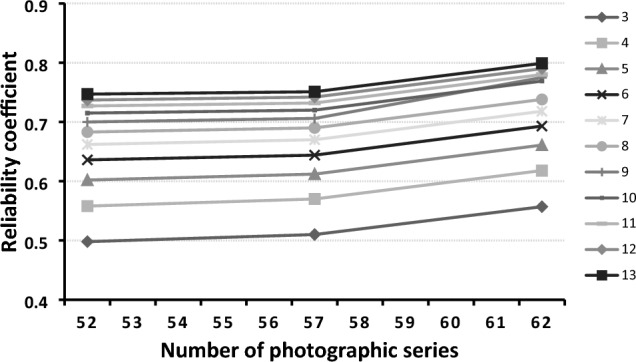
D-study for photographic assessment tool. The reliability coefficient (*y*-axis) is affected by the number of photographic series assessed (*x*-axis). Shaded lines represent a variable number of assessors (3–13). The critical G-coefficient of 0.8 was reached with 13 assessors each rating 62 photographic series (or 24 assessors each rating 52)

Generalizability analyses were performed to evaluate the reliability of the assessment tool with a fully crossed design using photographs (P), items (I) and assessors (A), such that (P × I × A)[19]. Raw scores of the 20-item photographic assessment tool were generalized over the assessor (A), and item (I). The overall reliability of the three assessors rating 62 photographic series each was represented by a generalizability coefficient of G(AI) = 0.557. D-studies were performed to examine the effect of increasing numbers of assessors (A) and photographic series (P) that they reviewed (Fig. 4).

### G-coefficients by assessors

During the analysis, assessor three was identified as a potential outlier. Therefore, separate G-coefficients were calculated for different combinations of assessors (A), see Table 1. This confirmed a lower G-coefficient when data from assessor three was utilized.

**Table 1:** G-coefficients by assessor combination

	A1 & A2	A1 & A3	A2 & A3
G-coefficient	0.599	0.305	0.570

### Inter-rater reliability and internal consistency

By setting the assessor as ‘random’ and as ‘A = 1’ while keeping the item as ‘fixed’, it was possible to generate the equivalent of inter-rater reliability as: Ep^2^ = 0.339. The equivalent of internal consistency could then be generated by setting the item as ‘random’ and the assessor as ‘fixed’: Ep^2^ = 0.873, which was similar to Cronbach alpha value: 0.869.

Through utilizing item-total statistics the Cronbach’s alpha reliability value could be calculated if individual assessment item were deleted. On review of item-total statistics for the 62 photographic data series, deletion of the overall assessment of the operation performed (D1, D1+ or D2), for each of the three raters, would cause an increased value of Cronbach’s alpha, see Table 2. As removal of this item would lead to a small improvement in the Cronbach’s alpha, and their respective ‘Corrected Item-Total Correlation’ values were low, this may lead one to consider removing these items from the photographic assessment tool. Cronbach alpha values and Corrected Item-Total Correlations did not differ significantly for other assessment items.

**Table 2:** Cronbach’s alpha item-total statistics

Task	Corrected item-total correlation	Cronbach’s alpha if item deleted
Distal Splenic Artery—Assessor 1	0.287	0.864
Distal Splenic Artery—Assessor 2	0.144	0.869
Distal Splenic Artery—Assessor 3	0.062	0.870
Splenic Vein Assessor 1	0.346	0.866
Splenic Vein Assessor 2	0.168	0.868
Splenic Vein Assessor 3	0.223	0.868
Assessor 1’s overall interpretation	-0.406	0.873
Assessor 2’s overall interpretation	-0.433	0.873
Assessor 3’s overall interpretation	-0.507	0.876

### Chi-square test of association

As removal of the assessors’ overall interpretation of the operation performed was shown to result in only small improvements in Cronbach’s alpha, a Chi-square test of association was used to discover if there was a relationship between assessors’ 19-item lymphadenectomy ratings versus their single-item global interpretation of the operation performed (D1, D1 + and D2), see Table 3. Intra-rater reliability was demonstrated for all assessors, as their lymphadenectomy ratings were significantly associated with their global interpretation of the operation performed. Phi and Cramer's V both demonstrated the highest strength of association in the ratings of assessors 1 and 3.

**Table 3:** Chi-square Test of Association between assessors’ 19-item lymphadenectomy rating and their single-item global interpretation of the operation performed

	Assessor 1	Assessor 2	Assessor 3
Pearson Chi-square	< 0.05	< 0.05	< 0.05
Phi	1.068	0.583	1.008
Crammer’s V	0.755	0.412	0.713

## Discussion

The principal outcome of this study was the development of a novel photographic assessment tool for SQA of gastrectomy. The tool was evaluated using data from the ADDICT Trial and is, therefore, well placed to support SQA in future clinical trials.

The development of the current tool was able to benefit from existing widely accepted descriptors for D1, D1+ and D2 gastrectomy as well as previous work relating to SQA in the context for oesophagectomy and colorectal surgery [9–12]. Use of photographic images for the purpose of SQA was noted to simplify the assessment process, albeit with some limitations that are commented on below. Furthermore, it was found that it was easier to capture, transfer and store still images compared to video files which have been utilized in previous SQA tools. Another strength of the proposed tool is its ability to allow the rater to determine not only the quality but also the extent of the lymphadenectomy (e.g., D1, D1+, D2) based on the remaining anatomy rather than the excised specimen. This has advantages in surgical RCTs, such as the ADDICT trial, where the differences between operative interventions must be clearly apparent to avoid a Type I or II error.

In this study, a photographic surgical quality assurance tool is used to reliably determine the extent of the lymphadenectomy performed during gastrectomy. Gastric lymph node stations have been comprehensively described and are universally accepted. While there may be variability in the volume of tissue and number of lymph nodes within individual lymph node stations, the anatomical structures that remain after their removal are clearly defined. The completeness of lymphadenectomy can, therefore, only be truly evaluated by reviewing the tissue and structures that remain and not by what is removed. The longstanding practice of using lymph node count as a surrogate of the completeness of lymphadenectomy, should not be considered a valid method of surgical quality assurance in this context.

This study nevertheless has several acknowledged limitations. While the importance of SQA as a concept is not in doubt, the template for its use is based on low-level evidence. Despite this, it has been replicated and published in upper and lower gastrointestinal surgical oncology trials with widespread surgeon acceptance and support for the framework described herein.

Including all three assessors and coding missing data as absent rather than a mean value [11], the G-coefficient and inter-rater reliability of the gastrectomy assessment tool fell below that attained previously with the esophagectomy tool in the ROMIO study [9]. However, the values obtained suggest that the tool can still be considered both valid and reliable.

The current study did highlight the importance of assessor selection and training with SQA tools. In this case, one assessor, despite being a consultant gastric cancer surgeon and demonstrating intra-rater reliability for the 19-item and single-item global rating scale, was the outlier in the group. It was noted that this assessor was the least experienced with SQA and had the longest lag time between the training session and their data submission. At present, seniority and familiarization with the operative intervention have been the major drivers in assessor selection. In future work, it may be necessary to demonstrate the same rigor in approving assessors for trial operative monitoring as there currently is for credentialing of surgeons for entry into a trial. In this research study, the three assessors guided the content of the tool and duration of training prior to using the tool, whereas a more formal approach and training strategy may be required to ensure objectivity and standardization of ratings. This improved approach is already being implemented in ongoing trials including the TIGER Study [23].

Beyond the SQA of gastrectomy as a fixed entity, being able to reliably assess the quality and extent of lymphadenectomy in gastric cancer resection offers the potential to definitively determine the relationship between radicality of lymphadenectomy and patient outcome. This could impact tailoring of individualized patient operations, reducing peri-operative morbidity from unnecessary dissection, improved survival, and prediction of disease recurrence patterns in those with lymph node involvement. The current study was not, however, designed or adequately powered to examine the link between SQA score and histopathological and clinical correlates. This has been identified as an important piece of further work that will be embedded within the ADDICT trial, to establish both the oncological and clinical validity of the tool.

Feedback received from all assessors was that short video clips of the operative field, at the end of the lymphadenectomy and post reconstruction, would potentially be more informative than still photographs (and longer unedited videos). It was felt that a dynamic view of the surgical field would help provide a more complete assessment of the resection through its ability to see structures from different angles. Furthermore, this could help to reduce missing data particularly that which is considered under the category of *insufficient evidence* which accounted for over 11% of data entry point in this study.

Future work will include examining the oncological clinical validity by correlating lymphadenectomy and tumor recurrence after completion of ADDICT. Adoption of artificial intelligence may also support real-time intra-operative guidance as well as automation of SQA processes. If achievable this could reduce assessors’ workload and improve reliability.

In conclusion, a novel photographic SQA tool is presented herein that may be used to assess the extent and quality of gastrectomy in the context of gastric cancer. The tool was determined to be objective and reliable, and allowed the assessor to evaluate not only the quality but also the extent of the lymphadenectomy. Assessor selection and training, however, remain central to ensuring the best performance of the tool.
