# Correction: Development of a reliable surgical quality assurance tool for gastrectomy in oncological trials

**DOI:** 10.1007/s10120-024-01547-w

**Published:** 2024-08-30

**Authors:** A. Harris, J. B. Butterworth, P. R. Boshier, S. Mavroveli, B. Vadhwana, C. J. Peters, B. W. Eom, C.-C. Yeh, S. Mikhail, M. Sasako, Y.-W. Kim, G. B. Hanna

**Affiliations:** 1 Department of Surgery and Cancer, Imperial College London, 7th Floor Commonwealth Building, Hammersmith Hospital, Du Cane Road, London, W12 0HS UK; 2 Department of Upper Gastrointestinal Surgery, Barking Havering and Redbridge University Hospitals NHS Trust, London, UK; 3 Center for Gastric Cancer, National Cancer Center, Seoul, Republic of Korea; 4 Department of Surgery, National Taiwan University Hospital, Taipei City, Taiwan; 5 Department of General Surgery, Cairo University, Cairo, Egypt; 6 Department of Surgery, Yodogawa Christian Hospital, Osaka, Japan

**Correction: Gastric Cancer (2024) 27:876–883** 10.1007/s10120-024-01503-8

The Funding information section was missing from this article and should have read “The research number (NCC-1410130, 1710120, 2010090, 2310210) for this study from National Cancer Center, Korea”.

The original article has been corrected.

